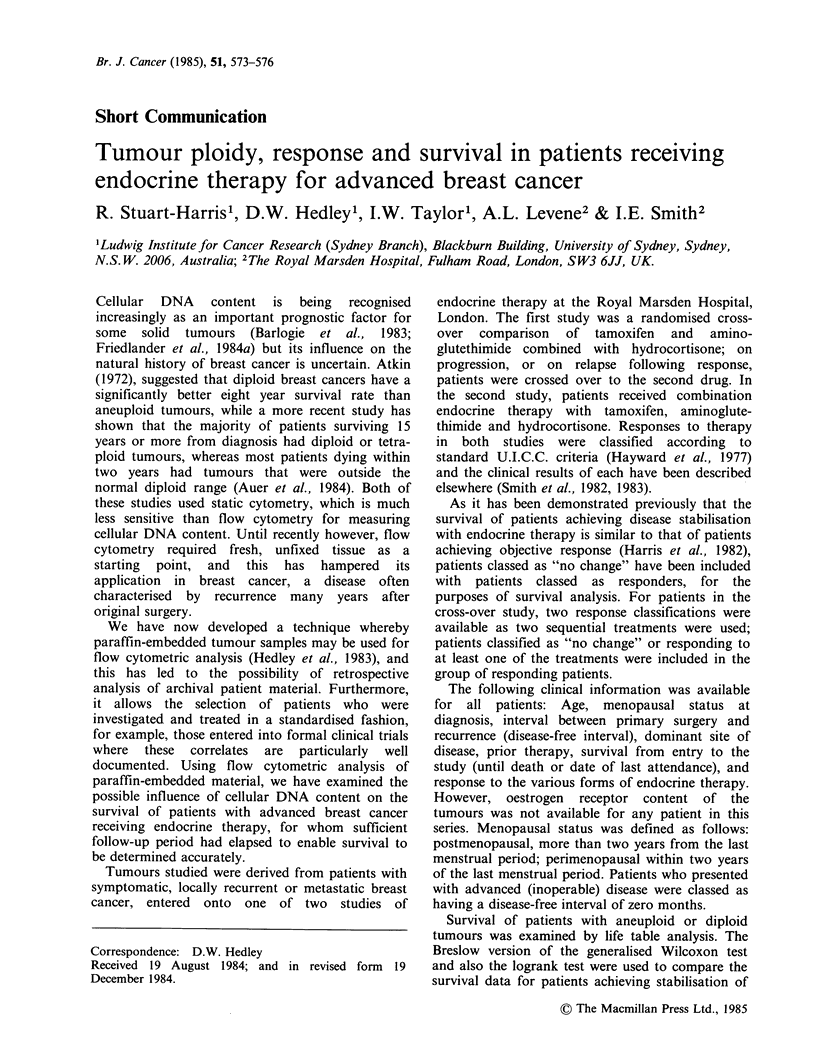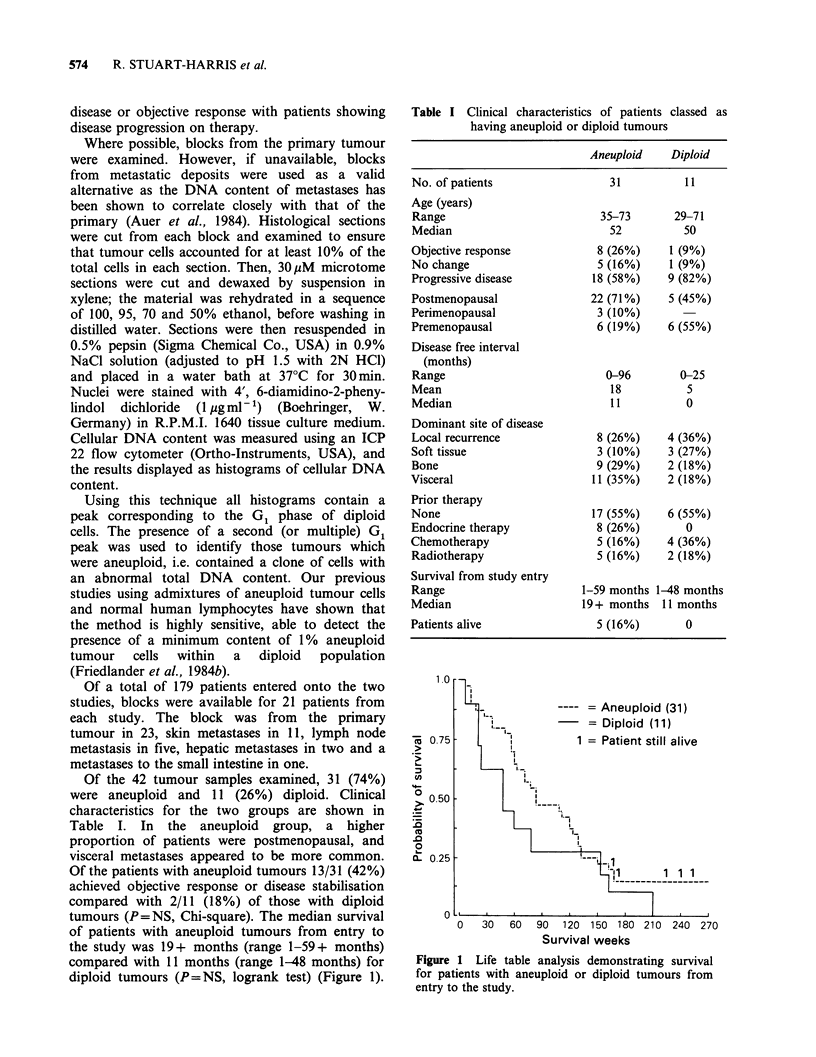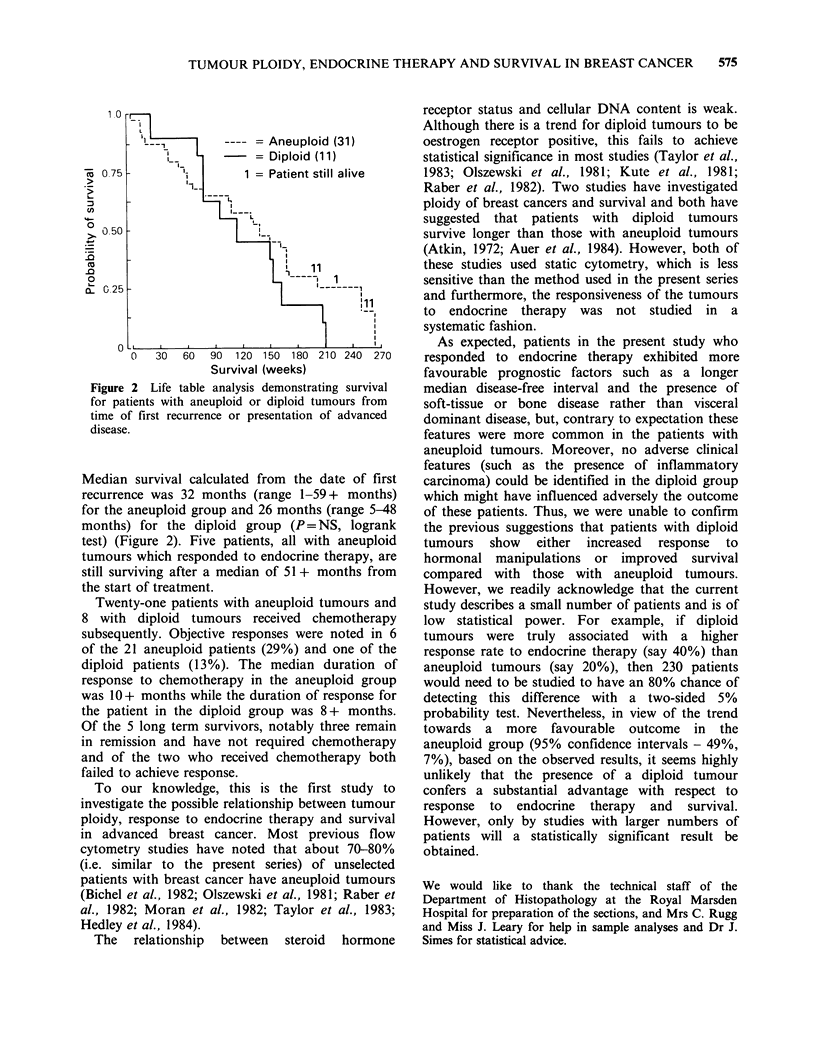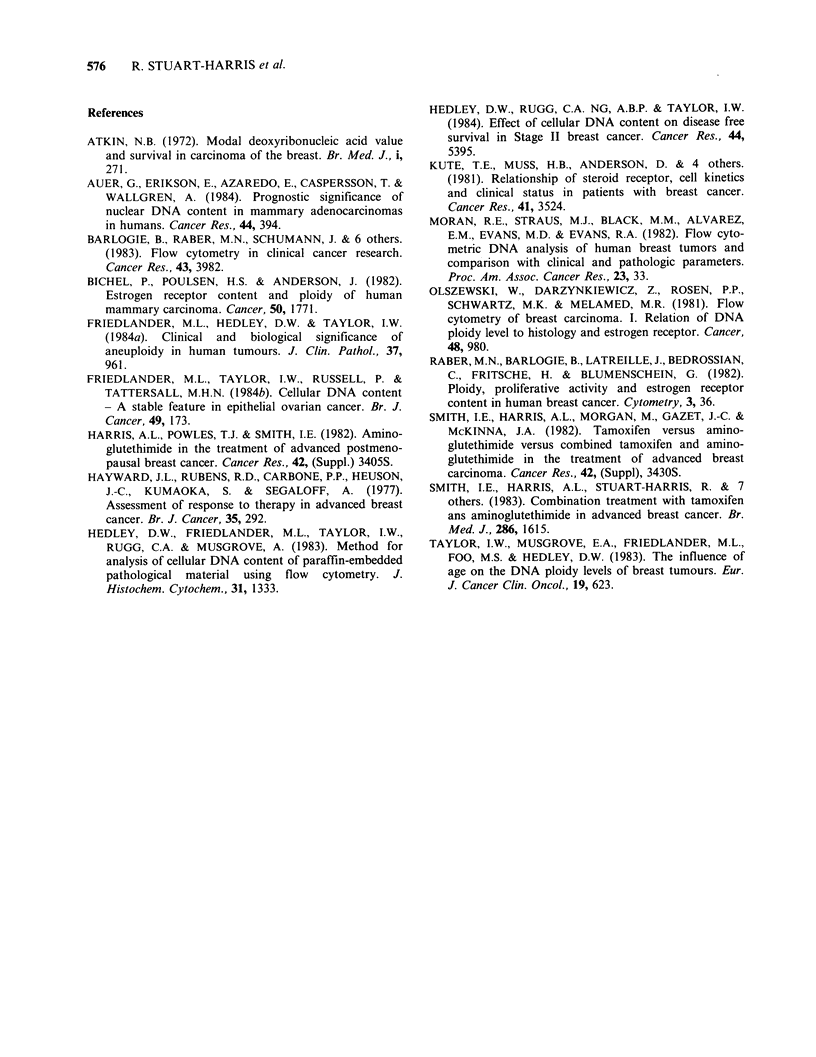# Tumour ploidy, response and survival in patients receiving endocrine therapy for advanced breast cancer.

**DOI:** 10.1038/bjc.1985.80

**Published:** 1985-04

**Authors:** R. Stuart-Harris, D. W. Hedley, I. W. Taylor, A. L. Levene, I. E. Smith


*Br. J. Cancer* (1985), **51**, 573–576

### Short Communication

### Tumour ploidy, response and survival in patients receiving endocrine therapy for advanced breast cancer

R. Stuart-Harris[1], D.W. Hedley[1], I.W. Taylor[1], A.L. Levene[2] & I.E. Smith[2]

[1]*Ludwig Institute for Cancer Research (Sydney Branch), Blackburn Building, University of Sydney, Sydney, N.S.W. 2006, Australia*; [2]*The Royal Marsden Hospital, Fulham Road, London, SW3 6JJ, UK.*

Cellular DNA content is being recognised increasingly as an important prognostic factor for some solid tumours (Barlogie *et al.*, 1983; Friedlander *et al.*, 1984*a*) but its influence on the natural history of breast cancer is uncertain. Atkin (1972), suggested that diploid breast cancers have a significantly better eight year survival rate than aneuploid tumours, while a more recent study has shown that the majority of patients surviving 15 years or more from diagnosis had diploid or tetraploid tumours, whereas most patients dying within two years had tumours that were outside the normal diploid range (Auer *et al.*, 1984). Both of these studies used static cytometry, which is much less sensitive than flow cytometry for measuring cellular DNA content. Until recently however, flow cytometry required fresh, unfixed tissue as a starting point, and this has hampered its application in breast cancer, a disease often characterised by recurrence many years after original surgery.

We have now developed a technique whereby paraffin-embedded tumour samples may be used for flow cytometric analysis (Hedley *et al.*, 1983), and this has led to the possibility of retrospective analysis of archival patient material. Furthermore, it allows the selection of patients who were investigated and treated in a standardised fashion, for example, those entered into formal clinical trials where these correlates are particularly well documented. Using flow cytometric analysis of paraffin-embedded material, we have examined the possible influence of cellular DNA content on the survival of patients with advanced breast cancer receiving endocrine therapy, for whom sufficient follow-up period had elapsed to enable survival to be determined accurately.

Tumours studied were derived from patients with symptomatic, locally recurrent or metastatic breast cancer, entered onto one of two studies of endocrine therapy at the Royal Marsden Hospital, London. The first study was a randomised cross-over comparison of tamoxifen and amino-glutethimide combined with hydrocortisone; on progression, or on relapse following response, patients were crossed over to the second drug. In the second study, patients received combination endocrine therapy with tamoxifen, aminoglutethimide and hydrocortisone. Responses to therapy in both studies were classified according to standard U.I.C.C. criteria (Hayward *et al.*, 1977) and the clinical results of each have been described elsewhere (Smith *et al.*, 1982, 1983).

As it has been demonstrated previously that the survival of patients achieving disease stabilisation with endocrine therapy is similar to that of patients achieving objective response (Harris *et al.*, 1982), patients classed as "no change" have been included with patients classed as responders, for the purposes of survival analysis. For patients in the cross-over study, two response classifications were available as two sequential treatments were used; patients classified as "no change" or responding to at least one of the treatments were included in the group of responding patients.

The following clinical information was available for all patients: Age, menopausal status at diagnosis, interval between primary surgery and recurrence (disease-free interval), dominant site of disease, prior therapy, survival from entry to the study (until death or date of last attendance), and response to the various forms of endocrine therapy. However, oestrogen receptor content of the tumours was not available for any patient in this series. Menopausal status was defined as follows: postmenopausal, more than two years from the last menstrual period; perimenopausal within two years of the last menstrual period. Patients who presented with advanced (inoperable) disease were classed as having a disease-free interval of zero months.

Survival of patients with aneuploid or diploid tumours was examined by life table analysis. The Breslow version of the generalised Wilcoxon test and also the logrank test were used to compare the survival data for patients achieving stabilisation of

Correspondence: D.W. Hedley
Received 19 August 1984; and in revised form 19 December 1984.

© The Macmillan Press Ltd., 1985

disease or objective response with patients showing disease progression on therapy.

Where possible, blocks from the primary tumour were examined. However, if unavailable, blocks from metastatic deposits were used as a valid alternative as the DNA content of metastases has been shown to correlate closely with that of the primary (Auer et al., 1984). Histological sections were cut from each block and examined to ensure that tumour cells accounted for at least 10% of the total cells in each section. Then, 30 $\mu$M microtome sections were cut and dewaxed by suspension in xylene; the material was rehydrated in a sequence of 100, 95, 70 and 50% ethanol, before washing in distilled water. Sections were then resuspended in 0.5% pepsin (Sigma Chemical Co., USA) in 0.9% NaCl solution (adjusted to pH 1.5 with 2N HCl) and placed in a water bath at 37°C for 30 min. Nuclei were stained with 4′, 6-diamidino-2-phenylindol dichloride (1 $\mu g\,ml^{-1}$) (Boehringer, W. Germany) in R.P.M.I. 1640 tissue culture medium. Cellular DNA content was measured using an ICP 22 flow cytometer (Ortho-Instruments, USA), and the results displayed as histograms of cellular DNA content.

Using this technique all histograms contain a peak corresponding to the $G_1$ phase of diploid cells. The presence of a second (or multiple) $G_1$ peak was used to identify those tumours which were aneuploid, i.e. contained a clone of cells with an abnormal total DNA content. Our previous studies using admixtures of aneuploid tumour cells and normal human lymphocytes have shown that the method is highly sensitive, able to detect the presence of a minimum content of 1% aneuploid tumour cells within a diploid population (Friedlander et al., 1984b).

Of a total of 179 patients entered onto the two studies, blocks were available for 21 patients from each study. The block was from the primary tumour in 23, skin metastases in 11, lymph node metastasis in five, hepatic metastases in two and a metastases to the small intestine in one.

Of the 42 tumour samples examined, 31 (74%) were aneuploid and 11 (26%) diploid. Clinical characteristics for the two groups are shown in Table I. In the aneuploid group, a higher proportion of patients were postmenopausal, and visceral metastases appeared to be more common. Of the patients with aneuploid tumours 13/31 (42%) achieved objective response or disease stabilisation compared with 2/11 (18%) of those with diploid tumours ($P$ = NS, Chi-square). The median survival of patients with aneuploid tumours from entry to the study was 19+ months (range 1–59+ months) compared with 11 months (range 1–48 months) for diploid tumours ($P$ = NS, logrank test) (Figure 1).

**Table I** Clinical characteristics of patients classed as having aneuploid or diploid tumours

| | *Aneuploid* | *Diploid* |
|---|---|---|
| No. of patients | 31 | 11 |
| Age (years) | | |
| Range | 35–73 | 29–71 |
| Median | 52 | 50 |
| Objective response | 8 (26%) | 1 (9%) |
| No change | 5 (16%) | 1 (9%) |
| Progressive disease | 18 (58%) | 9 (82%) |
| Postmenopausal | 22 (71%) | 5 (45%) |
| Perimenopausal | 3 (10%) | — |
| Premenopausal | 6 (19%) | 6 (55%) |
| Disease free interval (months) | | |
| Range | 0–96 | 0–25 |
| Mean | 18 | 5 |
| Median | 11 | 0 |
| Dominant site of disease | | |
| Local recurrence | 8 (26%) | 4 (36%) |
| Soft tissue | 3 (10%) | 3 (27%) |
| Bone | 9 (29%) | 2 (18%) |
| Visceral | 11 (35%) | 2 (18%) |
| Prior therapy | | |
| None | 17 (55%) | 6 (55%) |
| Endocrine therapy | 8 (26%) | 0 |
| Chemotherapy | 5 (16%) | 4 (36%) |
| Radiotherapy | 5 (16%) | 2 (18%) |
| Survival from study entry | | |
| Range | 1–59 months | 1–48 months |
| Median | 19+ months | 11 months |
| Patients alive | 5 (16%) | 0 |

**Figure 1** Life table analysis demonstrating survival for patients with aneuploid or diploid tumours from entry to the study.

**Figure 2** Life table analysis demonstrating survival for patients with aneuploid or diploid tumours from time of first recurrence or presentation of advanced disease.

Median survival calculated from the date of first recurrence was 32 months (range 1–59+ months) for the aneuploid group and 26 months (range 5–48 months) for the diploid group ($P$=NS, logrank test) (Figure 2). Five patients, all with aneuploid tumours which responded to endocrine therapy, are still surviving after a median of 51+ months from the start of treatment.

Twenty-one patients with aneuploid tumours and 8 with diploid tumours received chemotherapy subsequently. Objective responses were noted in 6 of the 21 aneuploid patients (29%) and one of the diploid patients (13%). The median duration of response to chemotherapy in the aneuploid group was 10+ months while the duration of response for the patient in the diploid group was 8+ months. Of the 5 long term survivors, notably three remain in remission and have not required chemotherapy and of the two who received chemotherapy both failed to achieve response.

To our knowledge, this is the first study to investigate the possible relationship between tumour ploidy, response to endocrine therapy and survival in advanced breast cancer. Most previous flow cytometry studies have noted that about 70–80% (i.e. similar to the present series) of unselected patients with breast cancer have aneuploid tumours (Bichel *et al.*, 1982; Olszewski *et al.*, 1981; Raber *et al.*, 1982; Moran *et al.*, 1982; Taylor *et al.*, 1983; Hedley *et al.*, 1984).

The relationship between steroid hormone receptor status and cellular DNA content is weak. Although there is a trend for diploid tumours to be oestrogen receptor positive, this fails to achieve statistical significance in most studies (Taylor *et al.*, 1983; Olszewski *et al.*, 1981; Kute *et al.*, 1981; Raber *et al.*, 1982). Two studies have investigated ploidy of breast cancers and survival and both have suggested that patients with diploid tumours survive longer than those with aneuploid tumours (Atkin, 1972; Auer *et al.*, 1984). However, both of these studies used static cytometry, which is less sensitive than the method used in the present series and furthermore, the responsiveness of the tumours to endocrine therapy was not studied in a systematic fashion.

As expected, patients in the present study who responded to endocrine therapy exhibited more favourable prognostic factors such as a longer median disease-free interval and the presence of soft-tissue or bone disease rather than visceral dominant disease, but, contrary to expectation these features were more common in the patients with aneuploid tumours. Moreover, no adverse clinical features (such as the presence of inflammatory carcinoma) could be identified in the diploid group which might have influenced adversely the outcome of these patients. Thus, we were unable to confirm the previous suggestions that patients with diploid tumours show either increased response to hormonal manipulations or improved survival compared with those with aneuploid tumours. However, we readily acknowledge that the current study describes a small number of patients and is of low statistical power. For example, if diploid tumours were truly associated with a higher response rate to endocrine therapy (say 40%) than aneuploid tumours (say 20%), then 230 patients would need to be studied to have an 80% chance of detecting this difference with a two-sided 5% probability test. Nevertheless, in view of the trend towards a more favourable outcome in the aneuploid group (95% confidence intervals – 49%, 7%), based on the observed results, it seems highly unlikely that the presence of a diploid tumour confers a substantial advantage with respect to response to endocrine therapy and survival. However, only by studies with larger numbers of patients will a statistically significant result be obtained.

We would like to thank the technical staff of the Department of Histopathology at the Royal Marsden Hospital for preparation of the sections, and Mrs C. Rugg and Miss J. Leary for help in sample analyses and Dr J. Simes for statistical advice.

### References

ATKIN, N.B. (1972). Modal deoxyribonucleic acid value and survival in carcinoma of the breast. *Br. Med. J.*, **i**, 271.

AUER, G., ERIKSON, E., AZAREDO, E., CASPERSSON, T. & WALLGREN, A. (1984). Prognostic significance of nuclear DNA content in mammary adenocarcinomas in humans. *Cancer Res.*, **44**, 394.

BARLOGIE, B., RABER, M.N., SCHUMANN, J. & 6 others. (1983). Flow cytometry in clinical cancer research. *Cancer Res.*, **43**, 3982.

BICHEL, P., POULSEN, H.S. & ANDERSON, J. (1982). Estrogen receptor content and ploidy of human mammary carcinoma. *Cancer*, **50**, 1771.

FRIEDLANDER, M.L., HEDLEY, D.W. & TAYLOR, I.W. (1984*a*). Clinical and biological significance of aneuploidy in human tumours. *J. Clin. Pathol.*, **37**, 961.

FRIEDLANDER, M.L., TAYLOR, I.W., RUSSELL, P. & TATTERSALL, M.H.N. (1984*b*). Cellular DNA content – A stable feature in epithelial ovarian cancer. *Br. J. Cancer*, **49**, 173.

HARRIS, A.L., POWLES, T.J. & SMITH, I.E. (1982). Aminoglutethimide in the treatment of advanced postmenopausal breast cancer. *Cancer Res.*, **42**, (Suppl.) 3405S.

HAYWARD, J.L., RUBENS, R.D., CARBONE, P.P., HEUSON, J.-C., KUMAOKA, S. & SEGALOFF, A. (1977). Assessment of response to therapy in advanced breast cancer. *Br. J. Cancer*, **35**, 292.

HEDLEY, D.W., FRIEDLANDER, M.L., TAYLOR, I.W., RUGG, C.A. & MUSGROVE, A. (1983). Method for analysis of cellular DNA content of paraffin-embedded pathological material using flow cytometry. *J. Histochem. Cytochem.*, **31**, 1333.

HEDLEY, D.W., RUGG, C.A. NG, A.B.P. & TAYLOR, I.W. (1984). Effect of cellular DNA content on disease free survival in Stage II breast cancer. *Cancer Res.*, **44**, 5395.

KUTE, T.E., MUSS, H.B., ANDERSON, D. & 4 others. (1981). Relationship of steroid receptor, cell kinetics and clinical status in patients with breast cancer. *Cancer Res.*, **41**, 3524.

MORAN, R.E., STRAUS, M.J., BLACK, M.M., ALVAREZ, E.M., EVANS, M.D. & EVANS, R.A. (1982). Flow cytometric DNA analysis of human breast tumors and comparison with clinical and pathologic parameters. *Proc. Am. Assoc. Cancer Res.*, **23**, 33.

OLSZEWSKI, W., DARZYNKIEWICZ, Z., ROSEN, P.P., SCHWARTZ, M.K. & MELAMED, M.R. (1981). Flow cytometry of breast carcinoma. I. Relation of DNA ploidy level to histology and estrogen receptor. *Cancer*, **48**, 980.

RABER, M.N., BARLOGIE, B., LATREILLE, J., BEDROSSIAN, C., FRITSCHE, H. & BLUMENSCHEIN, G. (1982). Ploidy, proliferative activity and estrogen receptor content in human breast cancer. *Cytometry*, **3**, 36.

SMITH, I.E., HARRIS, A.L., MORGAN, M., GAZET, J.-C. & McKINNA, J.A. (1982). Tamoxifen versus aminoglutethimide versus combined tamoxifen and aminoglutethimide in the treatment of advanced breast carcinoma. *Cancer Res.*, **42**, (Suppl), 3430S.

SMITH, I.E., HARRIS, A.L., STUART-HARRIS, R. & 7 others. (1983). Combination treatment with tamoxifen ans aminoglutethimide in advanced breast cancer. *Br. Med. J.*, **286**, 1615.

TAYLOR, I.W., MUSGROVE, E.A., FRIEDLANDER, M.L., FOO, M.S. & HEDLEY, D.W. (1983). The influence of age on the DNA ploidy levels of breast tumours. *Eur. J. Cancer Clin. Oncol.*, **19**, 623.